# Überraschung nach Treppensturz

**DOI:** 10.1007/s00347-020-01047-z

**Published:** 2020-02-07

**Authors:** Hanna Faber, Karl Ulrich Bartz-Schmidt, Jens Martin Rohrbach

**Affiliations:** Departement für Augenheilkunde, Universitätsklinikum Tübingen, Elfriede-Aulhorn-Str. 7, 72076 Tübingen, Deutschland

## Falldarstellung

**Figure 1:**
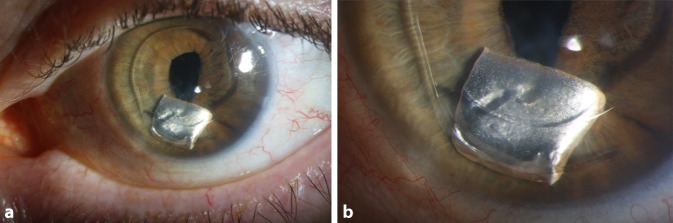

Ein 79-jähriger Patient stellte sich notfallmäßig wegen einer nach einem Treppensturz im linken Auge neu aufgetretenen spiegelnden Struktur in unserer Ambulanz vor. Er war zweieinhalb Wochen zuvor im Dunkeln gestürzt und zunächst notfallmäßig in der Urologie bei Verdacht auf Nierenblutung versorgt worden. Schmerzen am Auge wurden nicht angegeben. Der bestkorrigierte Visus des rechten Auges lag bei 1,0 und der des linken Auges bei Wahrnehmung von Handbewegungen. Der intraokulare Druck am rechten Auge lag bei 15 mmHg und am linken Auge bei 17 mmHg. Der vordere Augenabschnitt des rechten Auges war regelrecht. Am linken Auge zeigte sich in der Vorderkammer hinter der vor ca. 30 Jahren implantierten Vorderkammerlinse eine quadratische, silberfarbene Struktur (Abb. 1), welche vor dem Sturz nicht sichtbar gewesen war. Es bestand Aphakie, kein Vorderkammerreizzustand, allerdings eine beginnende Hornhautdekompensation.

## Wie lautet Ihre Diagnose?

## Therapie und Verlauf

Bei beginnender Hornhautdekompensation entschieden wir uns für die operative Entfernung des nichtmagnetischen intraokularen Fremdkörpers sowie der Vorderkammerlinse. Der intraoperative Magnetversuch war negativ. Daher erfolgte die Fremdkörperextraktion über einen korneoskleralen Stufenschnitt von 6 mm, über den auch die Vorderkammerlinse extrahiert werden konnte.

Intraoperativ konnte die Fremdkörperaufschlagstelle temporal unten auf der Netzhaut gesehen werden. Die Makula stellte sich unversehrt dar. Es zeigte sich eine Optikusatrophie. Postoperativ lag der bestkorrigierte Visus bei Handbewegungen.

Anamnestisch gab der Patient an, vor 50 Jahren während seiner Zeit bei der Bundeswehr eine Verletzung des Auges durch eine Explosion erlitten zu haben. Damals sei ein nichtmagnetischer Fremdkörper im Auge belassen worden.

## Diskussion

Intraokulare Fremdkörper im Rahmen von Traumata sind nicht selten. Im Gegensatz zu Hornhautfremdkörpern, die meist mit Photophobie, Epiphora, Blepharospasmus, Fremdkörpergefühl und Bindehautinjektionen einhergehen, können intraokulare Fremdkörper prinzipiell für lange Zeit unentdeckt bleiben.

Rezidivierende intraokulare Entzündungen bei gleichzeitiger Traumaanamnese sollten an einen verbliebenen intraokularen Fremdkörper denken lassen. Segi et al. berichten von einem 26-jährigen Mann mit 18 Jahre zurückliegendem okularem Trauma, der sich mit seit 3 Tagen bestehender Sehverschlechterung (Visus 0,5) und Schmerzen am rechten Auge vorstellte. Gonioskopisch konnte ein Fremdkörper im Kammerwinkel nachgewiesen werden und nach operativer Entfernung ein Visusanstieg auf 1,0 verzeichnet werden [3]. Bei Viestenz und Schönherr war ein 22 Jahre unbemerkt im Kammerwinkel liegender Glasfremdkörper für eine Hornhautdekompensation mit stark reduzierter Sehschärfe, Schmerzen und Blendungsempfindlichkeit verantwortlich [4].

**Diagnose:** Antero Mobilisation eines jahrzehntelang im Auge verbleibenden Fremdkörpers durch einen Sturz

Intraokulare Fremdkörper müssen nicht zwangsläufig zu Problemen führen. So zeigt nicht zuletzt die täglich durchgeführte Intraokularlinsenimplantation, dass das Auge intraokulare Fremdkörper problemlos über Jahre vertragen kann. Neuerungen der Kriegswaffen der letzten 100 Jahre gehen mit einem erhöhten Auftreten von Augenverletzungen im Rahmen kriegerischer Auseinandersetzungen einher [5]. Gleichzeitig haben Fortschritte der intraokularen Chirurgie, insbesondere die Entwicklung der Pars-plana-Vitrektomie die Versorgung traumatisierter Augen entscheidend verbessert. So wurde im Ersten Weltkrieg noch die Mehrzahl der verletzten Augen enukleiert [5]. Unser Fall lehrt, dass ein weniger radikales Vorgehen in bestimmten Fällen möglich ist und bestimmte intraokulare Fremdkörper prinzipiell problemlos im Auge verbleiben können. Bei Neumann et al. ging nur bei 1 von 10 Patienten ein verbliebener intraokularer, metallischen Fremdkörper mit einer Visusminderung einher [2]. Auch der vorliegende Fall zeigt, dass trotz aller Neuerung der Chirurgie vor jedem ophthalmochirurgischen Eingriff Nutzen und Risiko abgewogen werden müssen und im Zweifelsfall ein engmaschiges „abwartendes Beobachten“ auch eine Option sein könnte. Nichtsdestotrotz sollte heutzutage immer die Fremdkörperentfernung, insbesondere bei eisen- und kupferhaltigen sowie infizierten Fremdkörpern, angestrebt werden, da Erblindung droht [1]. Nur nicht infizierte und chemisch inerte FK, die sich nicht im Auge bewegen, können evtl. belassen werden. Die beginnende Hornhautdekompensation bei unserem Patienten war wahrscheinlich eher auf die Vorderkammerlinse als auf den (nicht frei flottierenden) Fremdkörper zurückzuführen.

## Fazit für die Praxis

Die ophthalmologische Anamnese sollte stets eine Traumaanamnese beinhalten. Intraokular verbliebene Fremdkörper können u. U. erst nach Jahren Beschwerden verursachen. Andererseits müssen als Zufallsbefund entdeckte Fremdkörper nur bei Hinweisen auf eine Augenschädigung, wie beispielsweise eine drohende Hornhautdekompensation, zwingend entfernt werden.
